# AML, NOS and AML-MRC as defined by multilineage dysplasia share a common mutation pattern which is distinct from AML-MRC as defined by MDS-related cytogenetics

**DOI:** 10.1038/s41375-022-01631-z

**Published:** 2022-06-20

**Authors:** Irene Fuhrmann, Miriam Lenk, Torsten Haferlach, Anna Stengel, Stephan Hutter, Constance Baer, Manja Meggendorfer, Wolfgang Kern, Claudia Haferlach

**Affiliations:** MLL Munich Leukemia Laboratory, Max-Lebsche-Platz 31, 81377 Munich, Germany

**Keywords:** Acute myeloid leukaemia, Cancer genetics

## To the Editor:

Acute myeloid leukemia (AML) is a genetically complex disease. Many different driver mutations are known to cause development of AML, moreover genetic and genomic features are the most important prognostic factors for overall survival [1]. In addition to cytogenetics, which have been pivotal in AML diagnostics and classification for many years, molecular genetics are becoming more important in everyday diagnostic routine.

According to WHO 2017 guidelines, AML cases without prior history of cytotoxic treatment are classified into subgroups with recurrent genetic abnormalities [2]. If these are not present, AML are either classified into the subgroup of AML with myelodysplasia-related changes (AML-MRC) or AML, not otherwise specified (AML, NOS). AML-MRC comprises patients with a history of myelodysplastic syndrome (MDS) or myelodysplastic/myeloproliferative neoplasm (MDS/MPN), or with multilineage myelodysplasia (MLD), or specific cytogenetic abnormalities. AML, NOS is subclassified based on morphological and cytochemical/immunophenotypic features of leukemic cells, indicating the lineages involved and their degree of maturation.

Since genetic aberrations are causal for development of AML, the classification on basis of specific gene mutations has the potential to divide patient groups according to biologic entities. However, knowledge about the genetic landscape of AML, NOS is scarce. Therefore, our aim was to evaluate the mutational pattern of AML, NOS and determine whether genetic features can help improve the classification of these patients. We compared these with the subgroup AML-MRC, which contains cases defined by cytogenetics, but also cases not defined by cytogenetics, possibly bearing resemblance to AML, NOS.

**Fig. 1:**
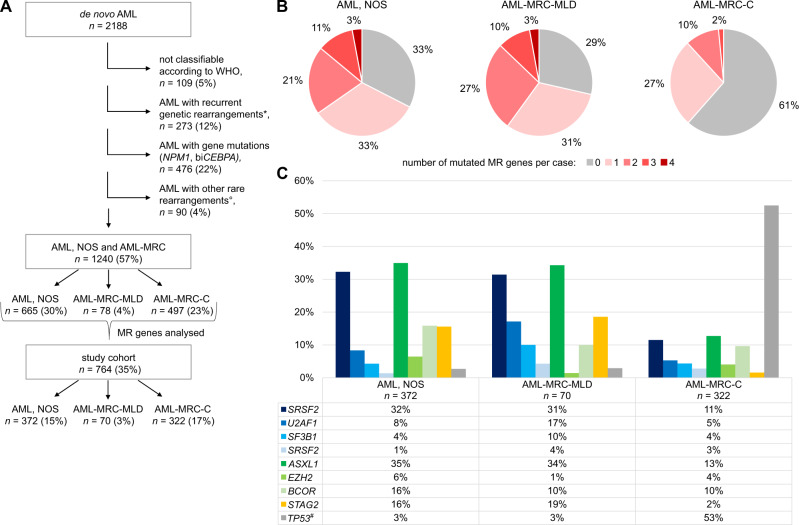
Overview, classification, and mutational analysis of the AML cases used in this study. **A** Breakdown of the AML cohort analysed in this study. 764 study cohort samples with molecular analyses were analysed in greater detail. Percentages refer to all de novo AML samples (*n* = 2188). *recurrent genetic rearrangements: *RUNX1*::*RUNX1T1, CBFB*::*MYH11, PML*::*RARA, KMT2A*::*MLLT3, DEK*::*NUP214, GATA2*::*MECOM, RBM15*::*MKL1, BCR*::*ABL1*. °other rare rearrangements: listed in Supplementary Table 1. bi*CEBPA*: biallelic-mutated *CEBPA*, AML-MRC-C: AML-MRC based on cytogenetics, AML-MRC-MLD: AML-MRC with isolated multilineage dysplasia, MR genes: myelodysplasia-related genes (*SRSF2, SF3B1, U2AF1, ZRSR2, ASXL1, EZH2, BCOR*, and *STAG2*). **B** Number of MR mutations per patient in the study cohort. Prevalence of these mutation numbers is shown for each group: AML, NOS (*n* = 372), AML-MRC-MLD (*n* = 70), and AML-MRC-C (*n* = 322). **C** Mutation rates of MR genes and *TP53* in the study cohort. Patient numbers for MR genes are given below the bar chart. Spliceosome genes are depicted in blue colours, chromatin modifying genes in green, cohesin component in yellow. ^#^data missing for 4 cases (AML, NOS: 1, AML-MRC-MLD: 1, AML-MRC-C: 2).

We retrospectively evaluated all newly diagnosed de novo AML cases between January 2018 and August 2021 (*n* = 2188), sent for routine diagnostics to MLL Munich Leukemia Laboratory. All patients had given written informed consent to the use of genetic and clinical data according to the Declaration of Helsinki. The study was approved by the internal review board. Cytomorphologic and cytogenetic analyses were performed as described before [3, 4]. Next generation sequencing was performed as described in the supplement. Of 2188 de novo AMLs, 109 (5%) were not classifiable according to WHO 2017 due to missing/insufficient bone marrow material or missing *NPM1* and/or *CEBPA* analyses in absence of recurrent genetic aberrations (Fig. 1A). Patients with fusion genes not defined by WHO were grouped in an additional “other rare rearrangements” group (details in Supplementary Table 1), since we aimed at analysing AML, NOS patients without any specific cytogenetic features. As *RUNX1* mutated AML is a provisional entity, *RUNX1* mutations were not used for classification and thus, according to the remaining classification criteria, all *RUNX1*-mutated cases were classified as AML, NOS. In summary, 273 cases (12%) were grouped into AML with recurrent genetic rearrangements, 476 cases (22%) into AML with gene mutations (*NPM1* or biallelic *CEBPA*), and 90 cases (4%) into other rare rearrangements. The remaining 1240 cases (cohort “AML, NOS and AML-MRC”, *n* = 1240, Fig. 1A) consisted of 497 (23%) cytogenetically-defined AML-MRC cases (AML-MRC-C), 78 (4%), AML-MRC defined by multilineage dysplasia (AML-MRC-MLD), and 665 patients (30%) with AML, NOS (detailed patient characteristics in Supplementary Table 1). The most prevalent karyotype in AML, NOS was a normal karyotype (70%), the most frequent chromosomal aberration was +8 (12%, Supplementary Fig. 1A, B).

**Fig. 2:**
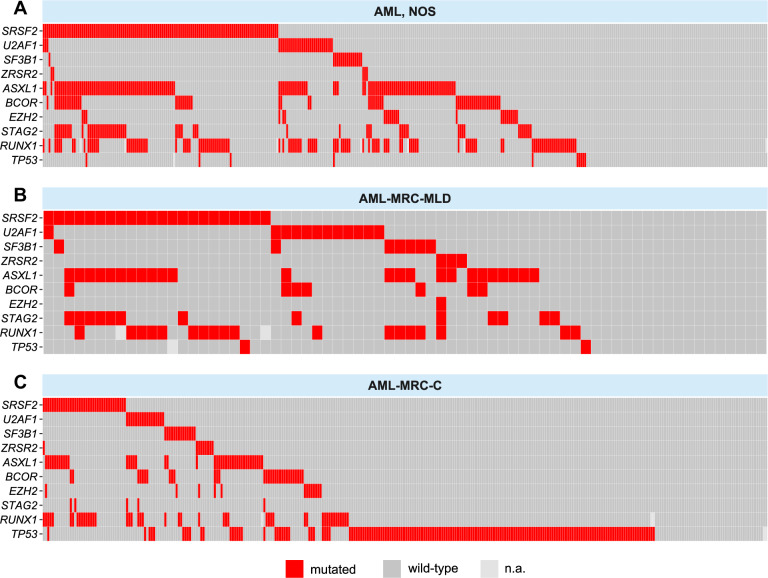
Mutation plots of study cohort patients. Visualisation of the mutational status of MR genes, *RUNX1*, and *TP53* in the AML subgroups (**A**) AML, NOS, **B** AML-MRC-MLD, and **C** AML-MRC-C. Each square represents one individual patient.

Inclusion criteria for an extensive examination of molecular patterns were mutation analyses of *SRSF2, SF3B1, U2AF1, ZRSR2, ASXL1, EZH2, BCOR*, and *STAG2* (hereafter named “MR genes” for myelodysplasia-related genes). These genes have been described by Lindsley et al. to be >95% specific for AML evolved from MDS or MDS/MPN [5]. The study cohort with cases having these analyses (*n* = 764; AML, NOS *n* = 372, AML-MRC-MLD *n* = 70, AML-MRC-C *n* = 322, Fig. 1A) did not differ from the cohort AML, NOS and AML-MRC regarding age, sex, bone marrow blasts, and blood counts (Supplementary Table 2A, B). Other gene mutation results were evaluated if available (details in Figs. 1C, 2 and Supplementary Fig. 3).

In AML, NOS, 67% of patients showed at least one MR mutation (Fig. 1B), in AML-MRC-MLD 71% of cases, respectively. In contrast, only 39% of patients in AML-MRC-C had one or more MR mutations (chi-square test, *p* < 0.001). Two or more MR mutations were found in 35% of AML, NOS, 40% of AML-MRC-MLD, but in only 8% of AML-MRC-C cases. The number of MR mutations per patient was significantly different between AML, NOS or AML-MRC-MLD on the one side and AML-MRC-C on the other side, but not between AML, NOS and AML-MRC-MLD (Welch ANOVA, Games-Howell post hoc test, *p* < 0.001): AML, NOS and AML-MRC-MLD cases showed a mean of 1.19 (median 1) and 1.27 (median 1) MR mutations per patient, whereas AML-MRC-C cases presented with a mean of 0.52 (median 0) MR mutations.

Mutation rates of specific MR genes varied substantially between the three subgroups. The strongest differences were observed for *SRSF2*: 32%, 31%, and 11%; *ASXL1:* 35%, 34%, and 13%; and *STAG2:* 16%, 19%, and 2%, in AML, NOS, AML-MRC-MLD, and AML-MRC-C, respectively (Fig. 1C). As expected, AML-MRC-C, encompassing cases with del(17p) and complex karyotype, had a higher prevalence of *TP53* mutations (53%) than AML, NOS and AML-MRC-MLD cases (3% each). In AML-MRC-C, the presence of *TP53* mutations and MR mutations was negatively correlated: *ASXL1, BCOR, EZH2, SRSF2*, and *U2AF1* showed significantly lower mutation rates when *TP53* was mutated compared to cases with wildtype *TP53* (chi-square test; *ASXL1*: *p* < 0.001, *BCOR: p* = 0.006, *EZH2: p* = 0.030, *SRSF2: p* < 0.001, *U2AF1: p* = 0.014, Supplementary Fig. 2). Mutation rates of *IDH1* and *IDH2* were higher in AML, NOS and AML-MRC-MLD than in AML-MRC-C (*IDH1*: 10%, 13%, and 7% and *IDH2*: 23%, 17%, and 11%, respectively, Supplementary Fig. 3). The same was true for mutation rates of *RUNX1* (32%, 26%, and 15%). The presence of *RUNX1* mutations was positively correlated with the presence of MR mutations in AML, NOS (chi-square test; *p* < 0.001): *RUNX1* mutations were twice as frequent in the presence of MR mutations compared to their absence (38% vs 19%, MR-mutated vs MR-wild-type AML, NOS). Only 23 cases (20% of all *RUNX1*-positive cases) were mutated in *RUNX1*, but negative for MR gene mutations.

We found a substantial part of AML, NOS and AML-MRC-MLD harbouring MR mutations (67% and 71%). A differentiation between AML, NOS cases with and without MR mutations has not yet been included in diagnostic and prognostic routines. Following WHO 2017, patients in AML, NOS are further subclassified according to morphology. These subtypes as well as subclassification according to the French-American-British (FAB) system did not show prognostic relevance [2, 6]. Likewise, the presence of MLD alone has no prognostic significance in AML-MRC [7].

Several studies have demonstrated that patients with de novo AML and MR mutations share the unfavourable clinical and prognostic features of AML progressed from MDS or MDS/MPN. Lindsley et al. showed these patients to be less responsive to chemotherapy [5]. Papaemmanuil et al. confirmed lower survival rates of patients with mutations of chromatin-spliceosome genes (e.g. *SRSF2*, *ASXL1*, *STAG2*) and found higher relapse rates compared to other subgroups [1]. A lower response rate and worse survival was corroborated for patients with mutations in *SRSF2, U2AF1*, and *ASXL1* by Gao et al. [8]. In line with these findings, Baer et al. identified a mutational pattern including *SRSF2*, *U2AF1*, *SF3B1*, *ASXL1*, *EZH2*, *BCOR*, and *STAG2* that allowed to distinguish AML-MRC from non-AML-MRC patients. Overall survival (OS) was inferior in patients harbouring these mutations, irrespective of whether they were classified as AML-MRC or not [9].

No detailed survival data was available for our consecutive and unselected study cohort. We therefore evaluated a second retrospectively assembled cohort (Supplementary Results, Supplementary Fig. 4). AML-MRC harbouring MR gene mutations and AML, NOS harbouring MR gene mutations showed a comparable OS, hinting at a shared characteristic of AML biology.

We found a high prevalence of MR mutations in AML, NOS. These were described to be specific for AML arising from a previous myeloid malignancy and result in a similar prognosis as that of AML-MRC patients. We believe that the presence of MR mutations reflects an underlying biology of AML. Therefore, we suggest to include an additional subgroup in AML-MRC, which is defined by molecular genetics, harbouring MR mutations (AML-MRC-M). Since prognostic relevance of MLD, if it is the only criterion for inclusion into AML-MRC, has not been shown, we suggest omitting cases with only MLD from the AML-MRC group. These cases would instead be classified as AML-MRC-M in presence of MR mutations and otherwise as AML, NOS. In our cohort, 50 out of 70 (71%) AML-MRC-MLD cases had MR mutations and would be re-classified to AML-MRC-M. The remaining 20 cases (29%) would become AML, NOS. In AML, NOS 251 out of 372 (67%) cases had MR mutations and would be re-classified to AML-MRC-M. The remaining 121 cases (33%) would remain AML, NOS. This would reduce the group of AML, NOS from originally 372 patients to 141 (38%, Supplementary Fig. 5).

In conclusion, patients with mutations in *SRSF2, SF3B1, U2AF1, ZRSR2, ASXL1, EZH2, BCOR*, or *STAG2* constitute a substantial proportion of patients diagnosed with AML, NOS or with AML-MRC-MLD. We suggest classifying cases with these mutations as a new subgroup of AML-MRC, likely reflecting the distinct biology of AML entities more accurately.

## Supplementary information

Supplementary text

Supplementary Figure 1

Supplementary Figure 2

Supplementary Figure 3

Supplementary Figure 4

Supplementary Figure 5

Supplementary Table 1

Supplementary Table 2

Supplementary Table 3
