# Supplementary material for: AML, NOS and AML-MRC as defined by multilineage dysplasia share a common mutation pattern which is distinct from AML-MRC as defined by MDS-related cytogenetics
Source: Leukemia. 2022 Jun 20;36(7):1939–42. doi: 10.1038/s41375-022-01631-z (PMC9252909; doi:10.1038/s41375-022-01631-z)
Supplement: Supplementary file 1 — Supplementary text [file 41375_2022_1631_MOESM1_ESM.docx]

**Supplemental Material**

**Supplementary Text**

**Methods**

**Molecular genetic analysis**

For DNA isolation, the MagNA Pure 96 system (Roche Diagnostics, Mannheim, Germany) was used. Sequencing was performed on NextSeq or MiSeq platforms after TruSeq Custom Amplicon Low Input library preparation or on NovaSeq after –Nextera DNA Flex library preparation (all Illumina, San Diego, CA) and enrichment with a custom panel by IDT (Integrated DNA Technologies, Iowa, USA) following manufacturer’s instructions. Gene variants were called either with SeqNext 4.3 (JSI Medical Systems, Kippenheim, Germany) or with Pisces (BaseSpace, Illumina), using a sensitivity level of 3%. For variant annotation several databases (COSMIC, IRAC and ClinVAR) and *in silico* predictions were used.

For mutation analysis only samples with a minimum read depth (coverage) of 400x for all protein-coding and intronic splice-donor/acceptor sites where used for genes *SRSF2*, *SF3B1*, *U2AF1*, *ZRSR2*, *ASXL1*, *EZH2*, *BCOR*, and *TP53.* For *STAG2* a coverage of 400x was required in >90% of all analyzed sites.

**Statistical analysis**

Statistical analyses were performed using SPSS software (version 19.0.0, IBM Corporation, Armonk, NY). All reported p-values are two-sided and were considered significant at p≤0.05. Overall survival was compared using log-rank test.

**Results**

**Overall survival analysis of second cohort**

Since our patients came from a real world consecutive and unselected cohort, no comprehensive survival data was available. We used a second, smaller, non-consecutive, and retrospectively assembled cohort of AML patients to analyse overall survival (OS) (n=231 for Supplementary Figure 4A+B+C, n=259 For Supplementary Figure 4D). Seven patients of the study cohort were also part of this second cohort.

Comparing patients with AML-MRC (n=84) and AML, NOS (n=147), following the same definition as used in this manuscript, adapted from current WHO classification, median OS was significantly shorter in patients with AML-MRC than in AML, NOS (114 days vs. 302 days, *p*=0.003, Supplementary Figure 4A). When further dividing the AML-MRC group into AML-MRC-C (n=78) and AML-MRC-MLD (n=6), the difference in median OS to AML, NOS was particularly pronounced for AML-MRC-C (88 days for AML-MRC-C, 263 days for AML-MRC-MLD, 302 days for AML, NOS, *p*=0.003, Supplementary Figure 4B).

Next, we applied our proposed classification to this cohort and categorised both AML, NOS and AML-MRC-MLD harbouring MR mutations into the AML-MRC-M group (n=102). AML-MRC-C remained the same, but was split into either patients with MR mutations (n=39) or *TP53* mutation (n=39). Median OS in the AML-MRC-M group (n=102, 433 days) seemed similar to median OS of AML-MRC-C patients with MR mutations (n=39, 358 days), whereas median OS of AML-MRC-C patients with *TP53* mutations was considerably shorter (n=39, 35 days, *p*<0.001, Supplementary Figure 4C). Thus, *TP53* mutations showed a greater impact on outcome than the presence of MR mutations, whereas patients with MR mutations had a similar survival, irrespective of the original subgroup according to WHO.

AML-MRC-C patients with both *TP53* and one or more MR mutations (n=8) and AML-MRC-C patients with neither *TP53* nor MR mutations (n=20) were at first excluded from the analysis due to the small number of cases. Including those cases in a final analysis, results have to be interpreted very carefully: MR mutations in addition to *TP53* mutations seemed to have little effect on survival compared to the 39 cases with only *TP53* mutations. Cases with neither *TP53* nor MR mutations seemed to fare slightly better (Supplementary Figure 4D).

**Supplementary Figures**

**Supplementary Figure 1 A+B**

Karyotypes of AML, NOS patients (grouped by morphology) and AML-MRC-MLD patients. (A) Percentage of patients with normal vs. aberrant karyotypes. (B) Aberrations found in cytogenetic analysis.

**Supplementary Figure 2**

Negative correlation of *TP53* and MR mutations in AML-MRC-C. Depicted are mutation rates of MR genes in dependence of *TP53* mutational status.

**Supplementary Figure 3 A-C**

Extensive mutation plots for patients with AML, NOS (A), AML-MRC-MLD (B), AML-MRC-C (C).

**Supplementary Figure 4 A-D**

Overall survival (OS) of AML patients in a second cohort, divided in different subgroups. Comparing AML-MRC and AML-NOS (A), AML-MRC-C, AML-MRC-MLD, and AML, NOS (B). Comparing AML-MRC-M and AML, NOS as defined by the suggested classification with AML-MRC-C with *TP53* mutations (AML-MRC-C TP53+) or MR gene mutations (AML-MRC-C MR+), (C). (A)-(C) exclude cases harbouring mutations in both *TP53* and MR genes (AML-MRC-C MR+TP53+) or without such mutations (AML-MRC-C double -). Final comparison as before, but including all six patient groups (AML-MRC-M, AML, NOS, AML-MRC-C TP53+, AML-MRC-C MR+, AML-MRC-C MR+TP53+, and AML-MRC-C double -) (D).

**Supplementary Figure 5**

Numbers of patients in the study cohort groups AML, NOS, AML-MRC-MLD, and AML-MRC-M as of now and if using MR mutations as group-defining factor.

**Supplementary Tables**

**Supplementary Table 1**

Detailed patient characteristics of all *de novo* AML cases.

**Supplementary Table 2 A+B**

Characteristics of patients with AML, NOS, AML-MRC-MLD, and AML-MRC-C.

Patient characteristics of cohort AML, NOS and AML-MRC (A, n=1,240) and study cohort cases (B, n=764). For all characteristics except sex, data is given as median (range). Not all variables were available for all patients. Differences within and between the cohorts were assessed using Pearson chi-square test (sex) and Welch ANOVA with Games-Howell post hoc test (all other variables). Results for group-wise post hoc comparisons are abbreviated as follows: NOS-MLD: AML, NOS vs AML-MRC-MLD, NOS-C: AML, NOS vs AML-MRC-C, MLD-C: AML-MRC-MLD vs AML-MRC-C, n.s.: not significant (p>0.05).

**Supplementary Table 3**

Analysis of overlap between MR mutations and *RUNX1* mutations in study cohort cases of AML, NOS.
