# Supplementary figures and images for: AML, NOS and AML-MRC as defined by multilineage dysplasia share a common mutation pattern which is distinct from AML-MRC as defined by MDS-related cytogenetics
Source: Leukemia. 2022 Jun 20;36(7):1939–42. doi: 10.1038/s41375-022-01631-z (PMC9252909; doi:10.1038/s41375-022-01631-z)

**A**

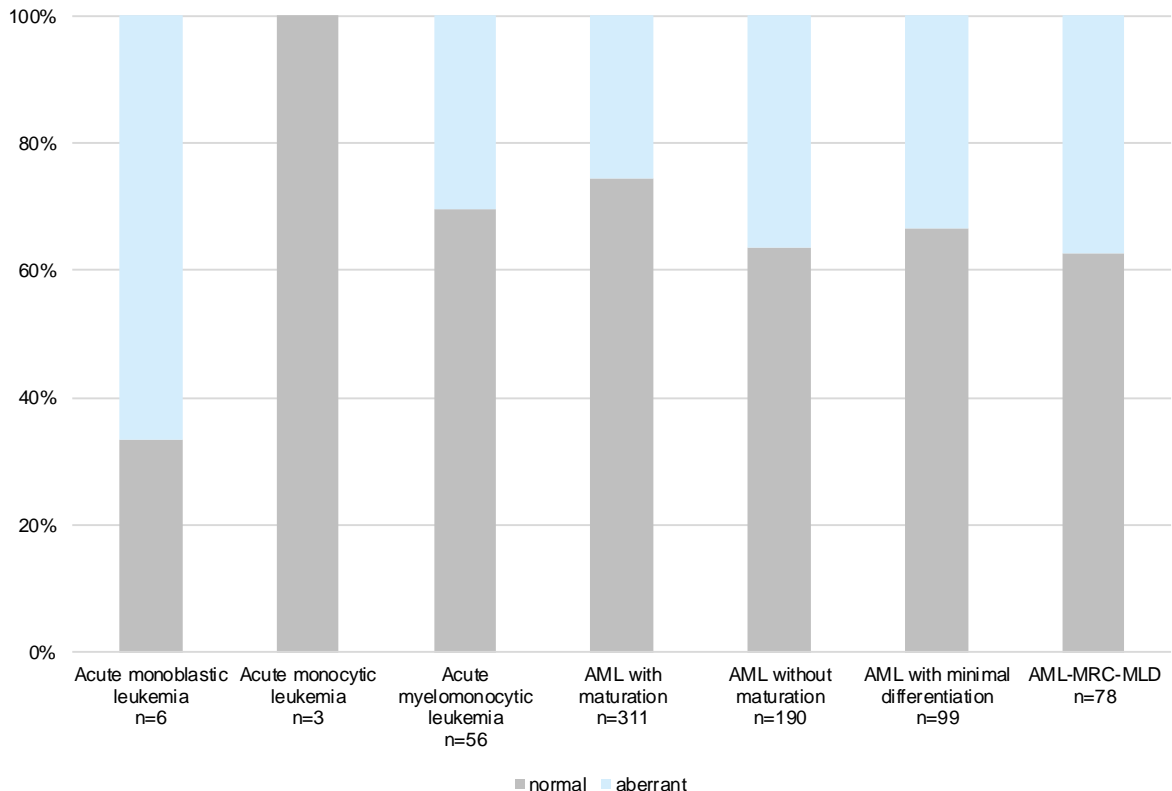

**B**

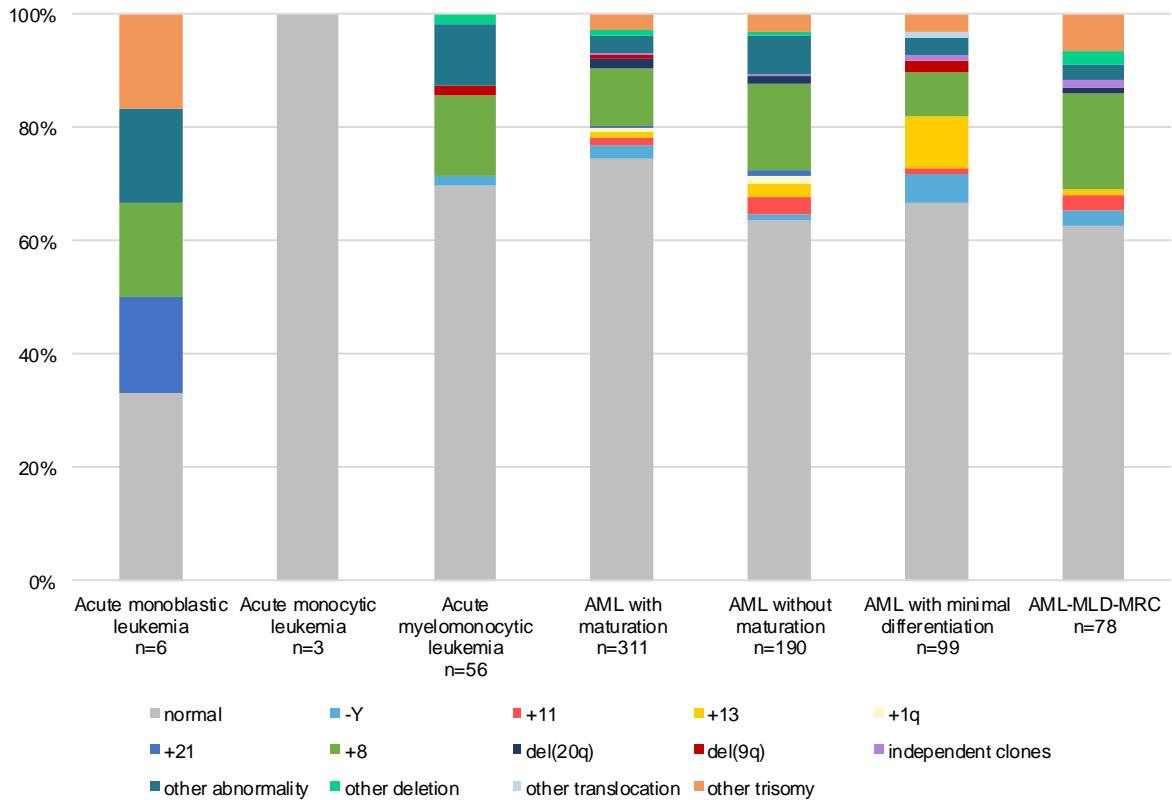

Supplement: Supplementary file 2 — Supplementary Figure 1 [file 41375_2022_1631_MOESM2_ESM.pdf]

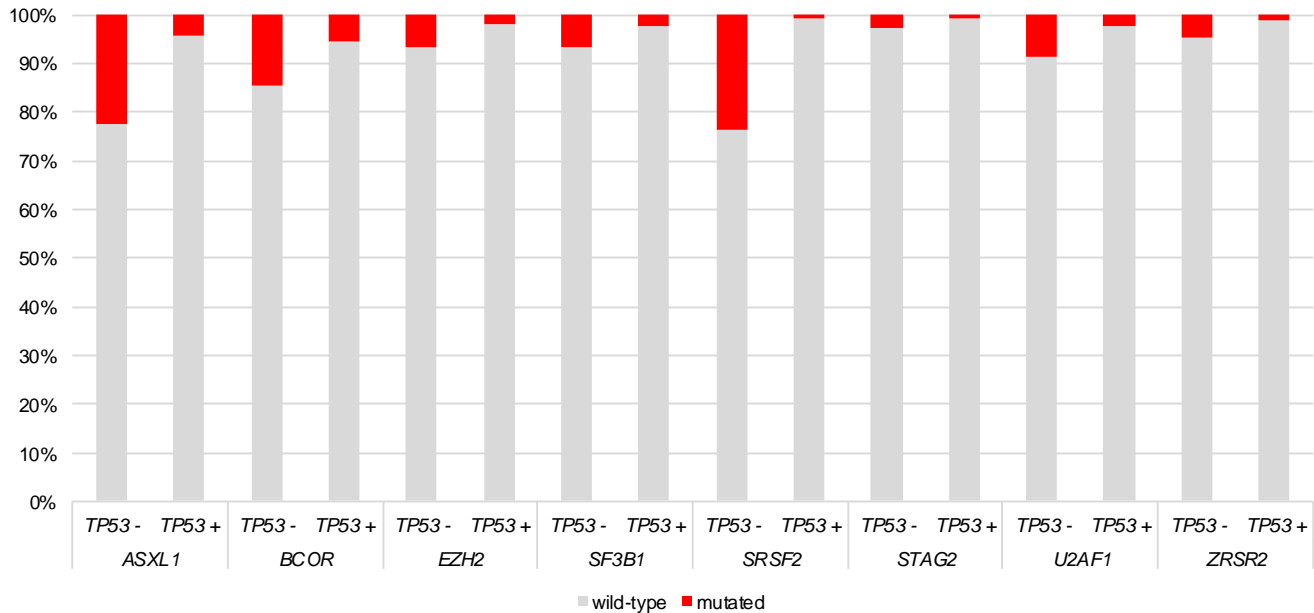

Supplement: Supplementary file 3 — Supplementary Figure 2 [file 41375_2022_1631_MOESM3_ESM.pdf]

A

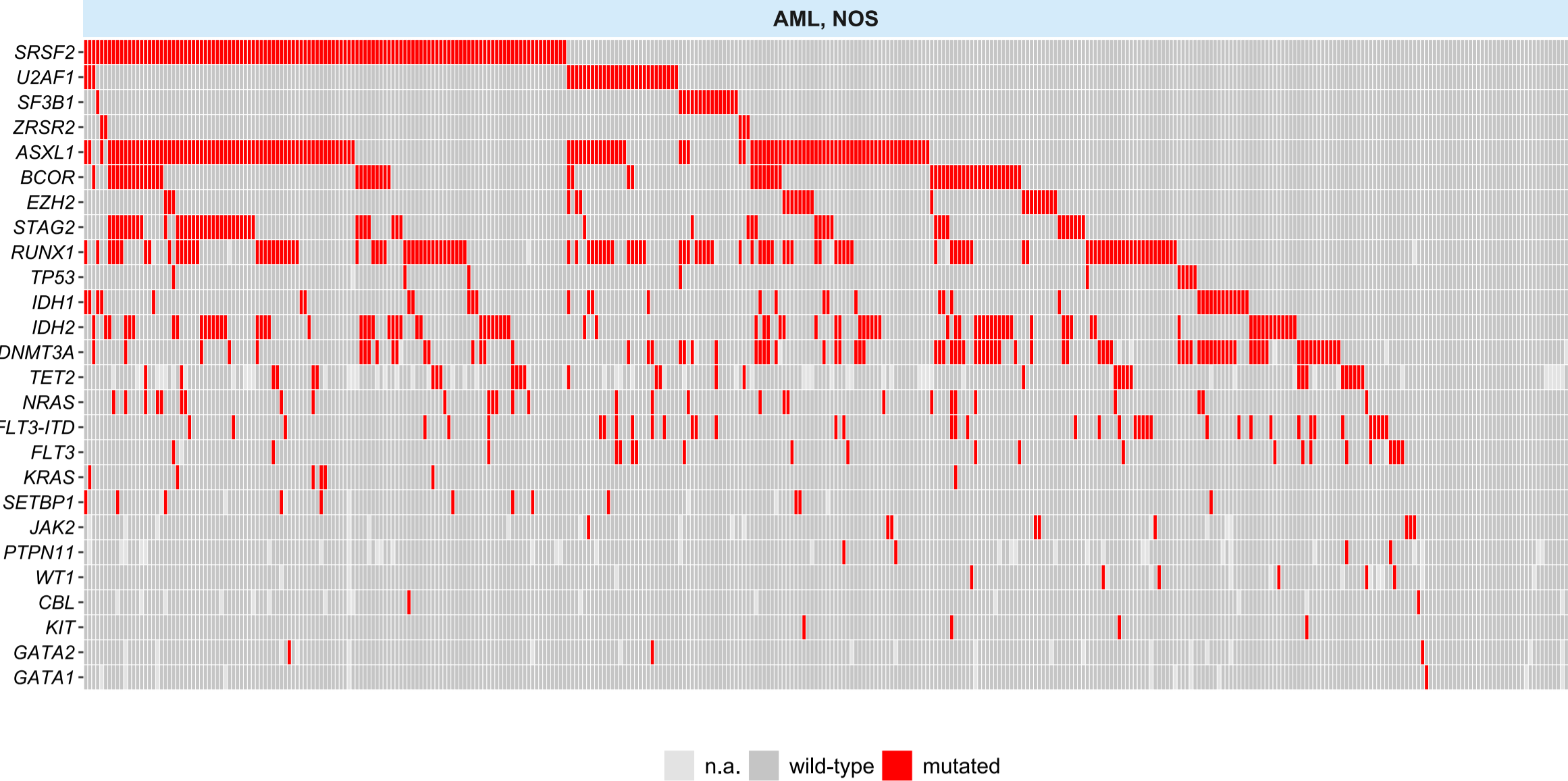

B

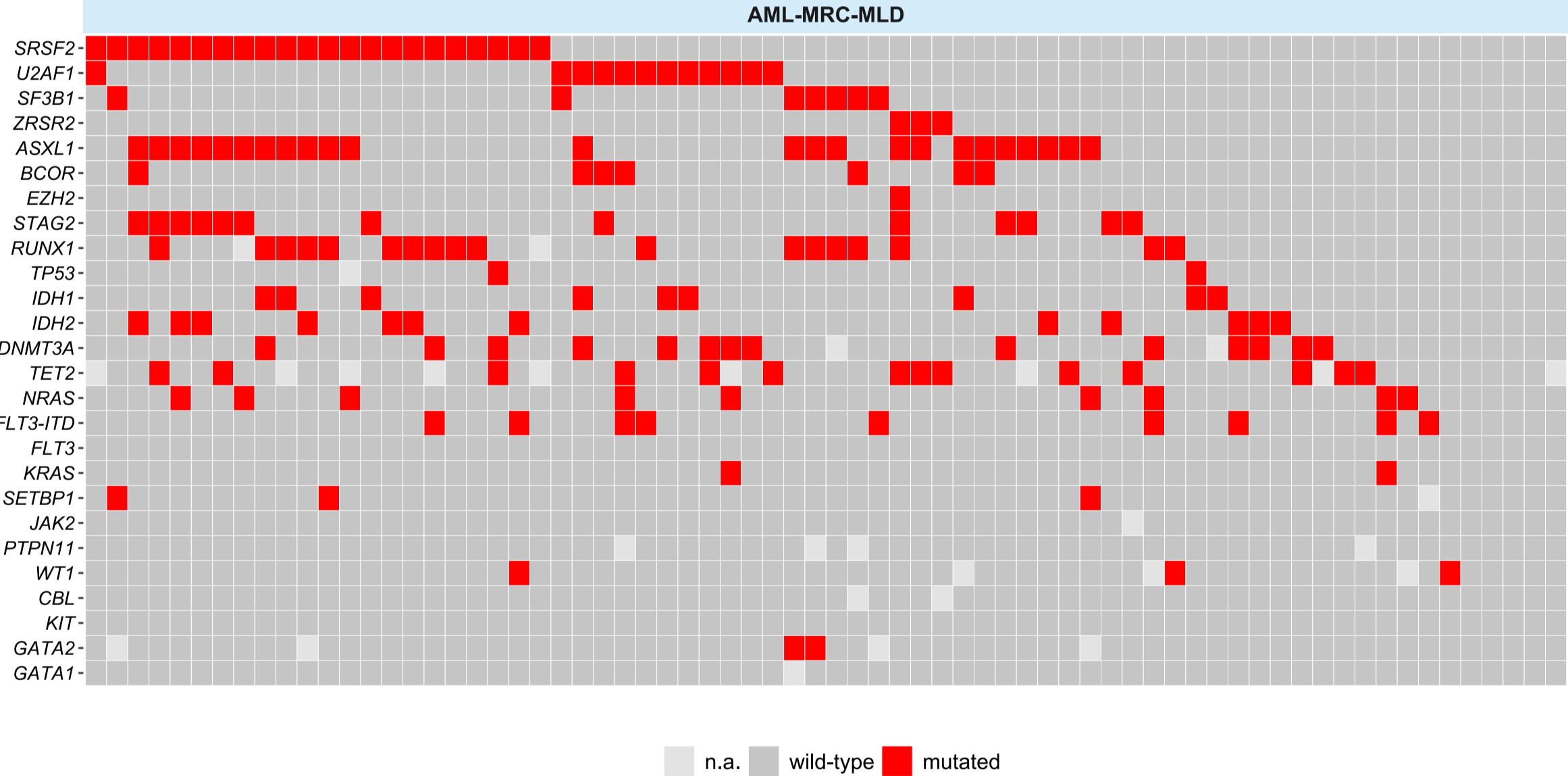

C

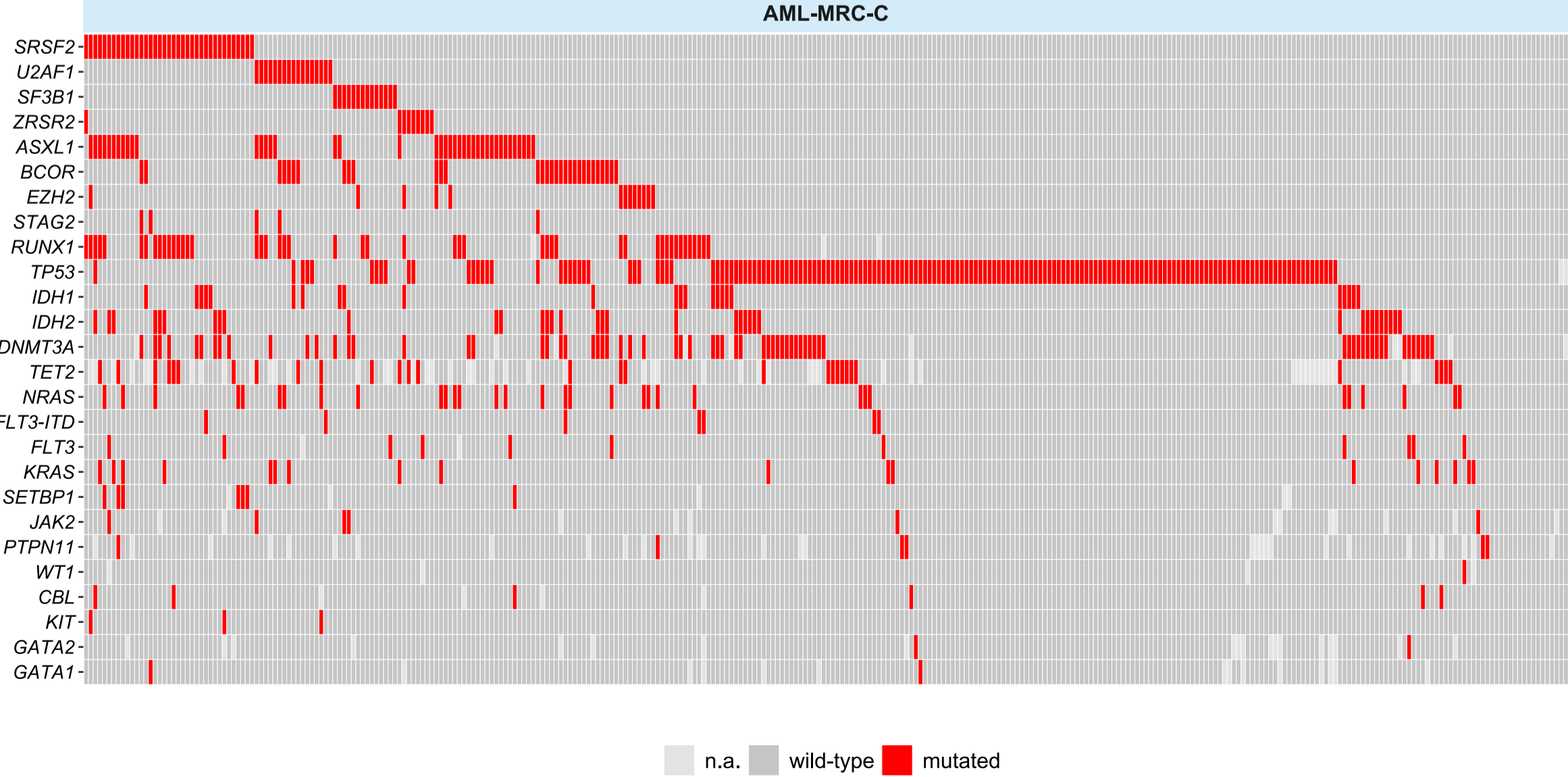

Supplement: Supplementary file 4 — Supplementary Figure 3 [file 41375_2022_1631_MOESM4_ESM.pdf]

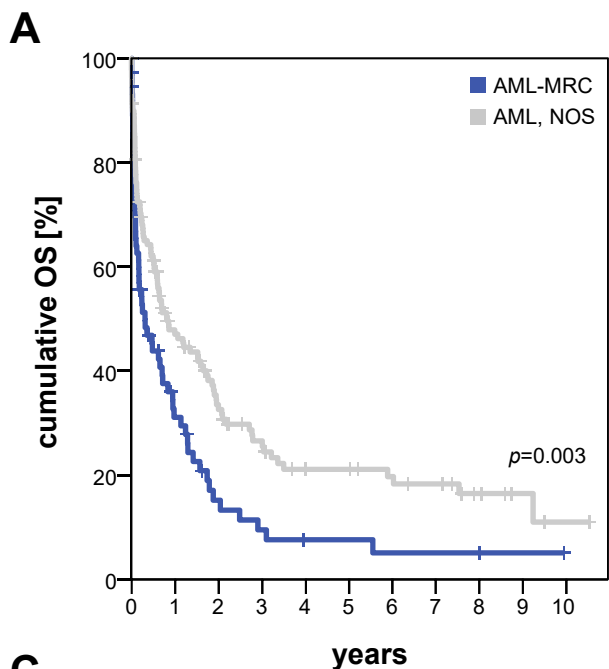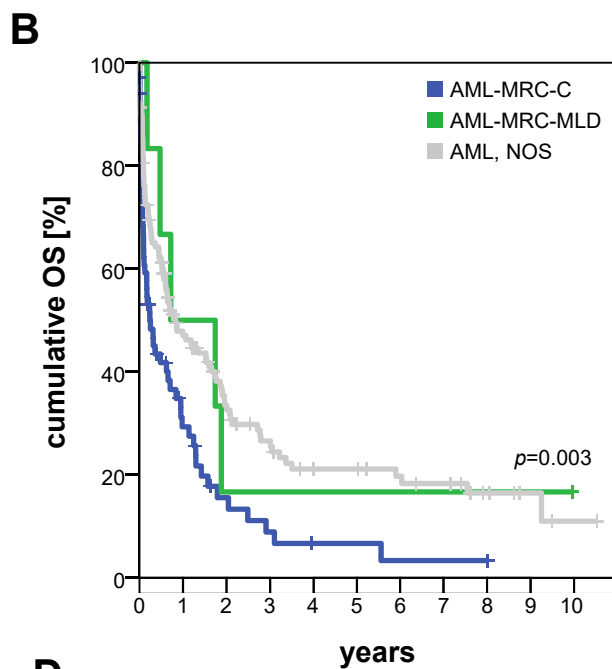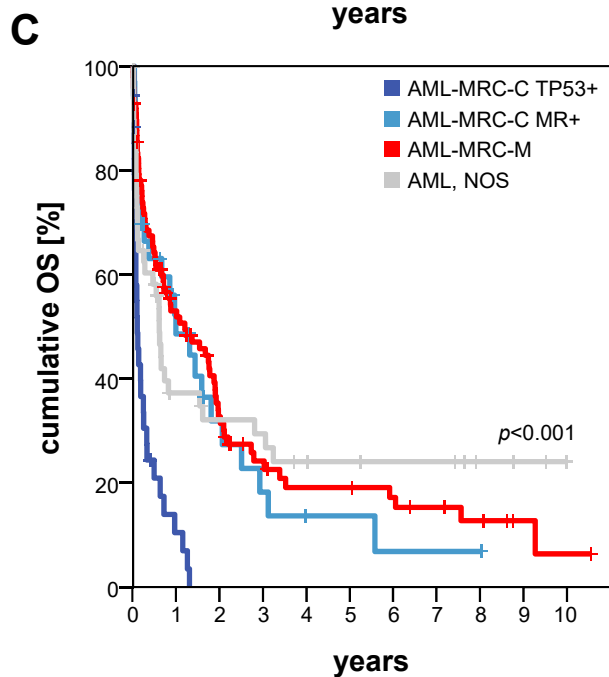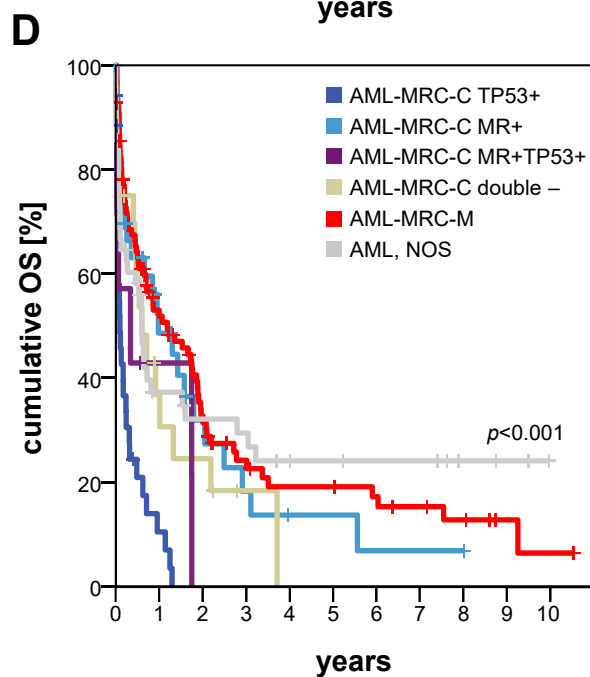

Supplement: Supplementary file 5 — Supplementary Figure 4 [file 41375_2022_1631_MOESM5_ESM.pdf]

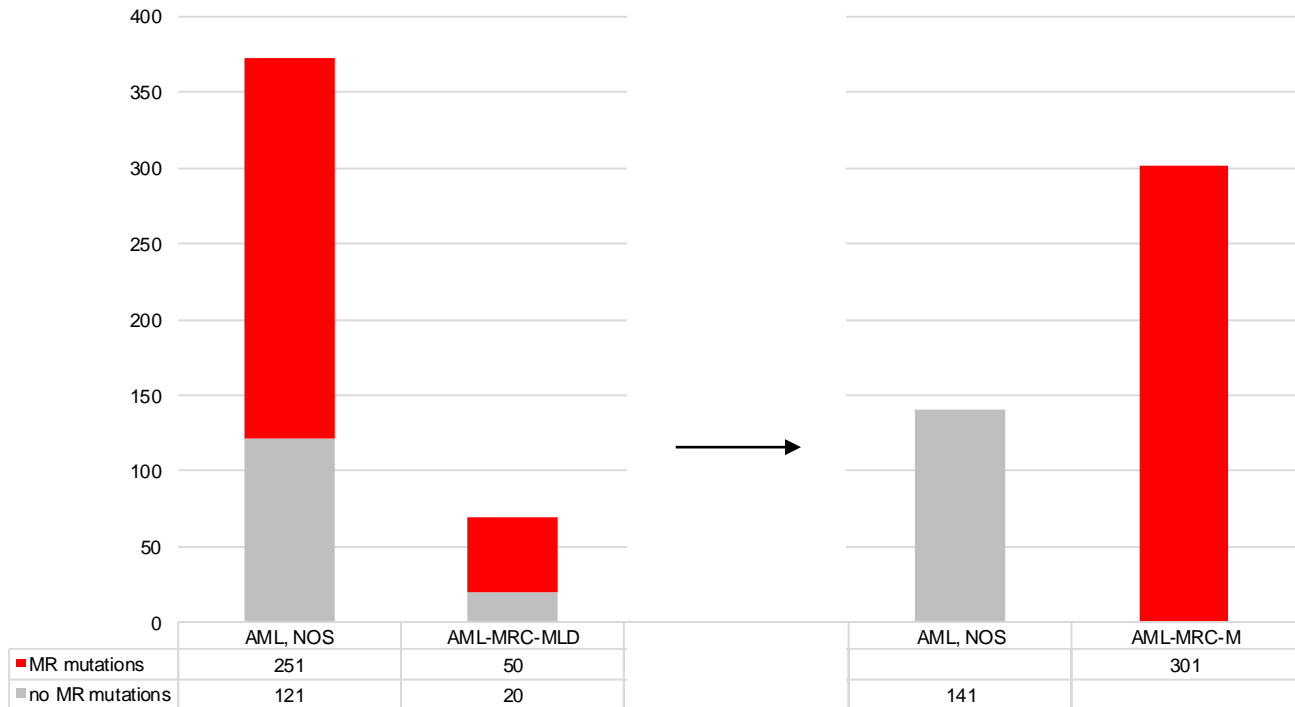

Supplement: Supplementary file 6 — Supplementary Figure 5 [file 41375_2022_1631_MOESM6_ESM.pdf]
